# Epigenetics in thyroid cancer: a bibliometric analysis

**DOI:** 10.1530/EC-24-0087

**Published:** 2024-08-07

**Authors:** Hui Li, Peng Wu

**Affiliations:** 1 Department of Thyroid Surgery, The Affiliated Cancer Hospital of Xiangya School of Medicine, Central South University/Hunan Cancer Hospital, Changsha, Hunan, P. R. China.

**Keywords:** bibliometric analysis, CiteSpace, epigenetics, thyroid cancer, VOSviewer

## Abstract

**Background:**

Epigenetics, which involves regulatory modifications that do not alter the DNA sequence itself, is crucial in the development and progression of thyroid cancer. This study aims to provide a comprehensive analysis of the epigenetic research landscape in thyroid cancer, highlighting current trends, major research areas, and potential future directions.

**Methods:**

A bibliometric analysis was performed using data from the Web of Science Core Collection (WOSCC) up to 1 November 2023. Analytical tools such as VOSviewer, CiteSpace, and the R package ‘bibliometrix’ were employed for comprehensive data analysis and visualization. This process identified principal research themes, along with influential authors, institutions, and countries contributing to the field.

**Results:**

The analysis reveals a marked increase in thyroid cancer epigenetics research over the past two decades. Emergent key themes include the exploration of molecular mechanisms and biomarkers, various subtypes of thyroid cancer, implications for therapeutic interventions, advancements in technologies and methodologies, and the scope of translational research. Research hotspots within these themes highlight intensive areas of study and the potential for significant breakthroughs.

**Conclusion:**

This study presents an in-depth overview of the current state of epigenetics in thyroid cancer research. It underscores the potential of epigenetic strategies as viable therapeutic options and provides valuable insights for researchers and clinicians in advancing the understanding and treatment of this complex disease. Future research is vital to fully leverage the therapeutic possibilities offered by epigenetics in the management of thyroid cancer.

## Introduction

Epigenetic regulation, which involves reversible and heritable changes in gene expression that occur in response to external stimuli without altering the DNA sequence (1, 2), is fundamental to understanding the pathogenesis of various diseases, including thyroid cancer (3, 4). This regulation includes DNA methylation, chromatin remodeling, histone modifications, and the expression of diverse non-coding RNAs (ncRNAs), such as microRNAs (miRNAs), long ncRNAs (lncRNAs), and circular RNAs (circRNAs) (5, 6, 7). These epigenetic processes are essential for elucidating the intricate mechanisms underlying the development and progression of thyroid cancer. Furthermore, the study of these epigenetic pathways holds significant promise for the identification of novel diagnostic, prognostic, and therapeutic targets in the management of thyroid cancer (8, 9, 10).

The growing interest in the role of epigenetics in thyroid cancer is evident from the rising number of publications. However, a comprehensive bibliometric analysis of this body of work is lacking. Such an analysis is vital for discerning publication trends, key contributors, and emerging research areas, thereby offering a more profound understanding of the field and highlighting areas of future interest (11, 12).

This study presents a detailed bibliometric analysis of scientific literature on epigenetics in thyroid cancer from 2013 to 2023, utilizing data from the WOSCC. Our goal is to provide an extensive overview of research output, collaborations, key authors and institutions, and emerging trends. This analysis will enhance understanding of the current research landscape in this area and guide future investigations.

## Materials and methods

### Search strategy

**Figure 1:**
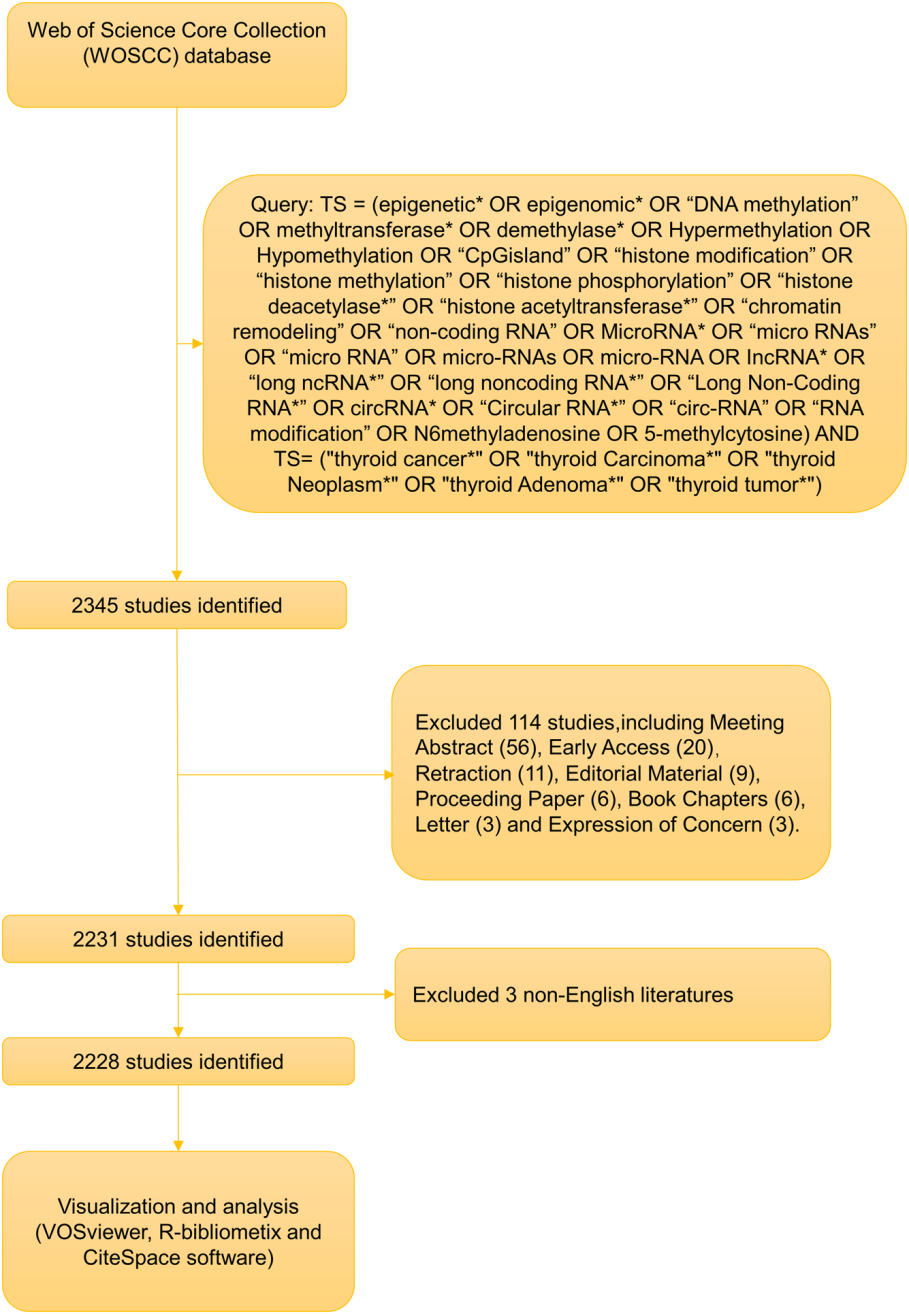
Screening process for literature selection in the study.

A comprehensive literature search was conducted from 1 January 2013 to 1 November 2023. To ensure thoroughness and accuracy, two independent reviewers performed the search, with any differences resolved through discussion or third-party consultation. The complete dataset was acquired in TEXT format on 15 November 2023. In conducting our bibliometric analysis, we established specific inclusion and exclusion criteria to ensure the relevance and quality of the data. Inclusion criteria: we included peer-reviewed research articles and reviews published in English, focusing on the epigenetics of thyroid cancer. Exclusion criteria: we excluded meeting abstracts, editorials, and other non-peer-reviewed publications to maintain scientific rigor. Additionally, publications not directly related to the specific focus of thyroid cancer epigenetics were omitted. All literature included in this analysis was sourced from the WOSCC, covering a period from 1 January 2013 to 1 November 2023. A detailed representation of our data collection and analysis methodology is shown in Fig. 1.

### Data analysis

In our bibliometric analysis, we utilized specific software tools known for their robust methodologies in examining scholarly data:

1. VOSviewer (version 1.6.19): Developed by the Centre for Science and Technology Studies at Leiden University in the Netherlands, VOSviewer is a freely available tool designed for constructing and visualizing bibliometric networks (13). It is instrumental for constructing and visualizing bibliometric networks, such as collaboration networks, co-citation analyses, and keyword co-occurrence analyses (14). This tool facilitates the identification of key patterns and trends within large publication datasets, allowing us to visualize the strength and scope of academic collaborations and the evolution of research themes within the field of epigenetics in thyroid cancer (15, 16).
2. CiteSpace (version 6.2.R4): Developed by Dr Chaomei Chen at Drexel University in Philadelphia, USA, CiteSpace is a Java application available under a freeware license (17). It is renowned for its advanced capabilities in bibliometric analysis, particularly for creating dual-map overlays and conducting in-depth cluster analyses of references and keywords (18, 19, 20). The CiteSpace software utilizes several structural metrics for analyzing co-citation networks, including betweenness centrality, modularity, and silhouette (21). Betweenness centrality quantifies the extent to which a node acts as a bridge along the shortest path between two other nodes within the network. Nodes with high betweenness centrality are often key to identifying influential scientific literature. Modularity (*Q*) evaluates the strength of division within a network into distinct communities or modules, with values ranging from 0 to 1. Typically, a *Q* value greater than 0.3 indicates a significant division in community structure. The mean silhouette (*S*) metric assesses the coherence of nodes within their clusters versus their placement on the margins between clusters; generally, an S value above 0.7 indicates a high degree of cohesion within a cluster, while a score above 0.5 suggests that the clustering is credible (22).
3. Bibliometrix R package (version 3.2.1): Bibliometrix is an R package developed for comprehensive bibliometric analysis of scientific literature (23). This open-source tool supports a range of quantitative research activities in bibliometrics, including data processing, descriptive analysis, and network analysis. It generates essential metrics such as citation counts, collaboration indices, and impact measures, which are instrumental in developing a detailed understanding of the dynamics within research domains. Additionally, Bibliometrix assesses journal impact factors and categorizes them based on the latest Journal Citation Reports (JCR) (24).

These tools were employed not only for their ability to handle large volumes of bibliographic data but also for their specific analytical capabilities that enable a deeper understanding of the patterns, trends, and structural relationships within the epigenetics of thyroid cancer literature.

## Results

### Publication trends in thyroid cancer epigenetics research

**Figure 2:**
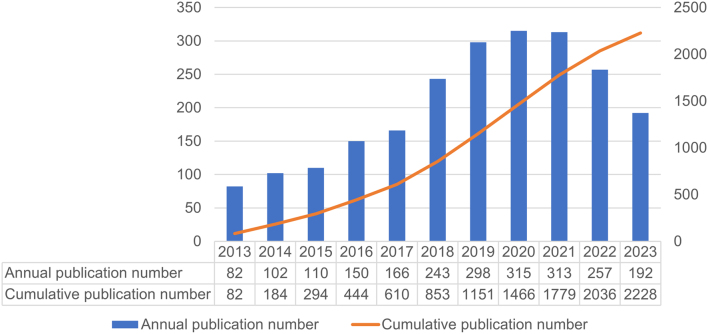
Annual trends in research output for epigenetics in thyroid cancer.

Our bibliometric analysis identified 2228 studies on epigenetics in thyroid cancer published from 2013 to 1 November 2023, including 1882 articles and 346 reviews. An upward trend in annual publications was observed, reaching a peak in 2020 before a slight decline, yet maintaining high overall numbers (Fig. 2).

### Geographical and institutional distribution

**Figure 3:**
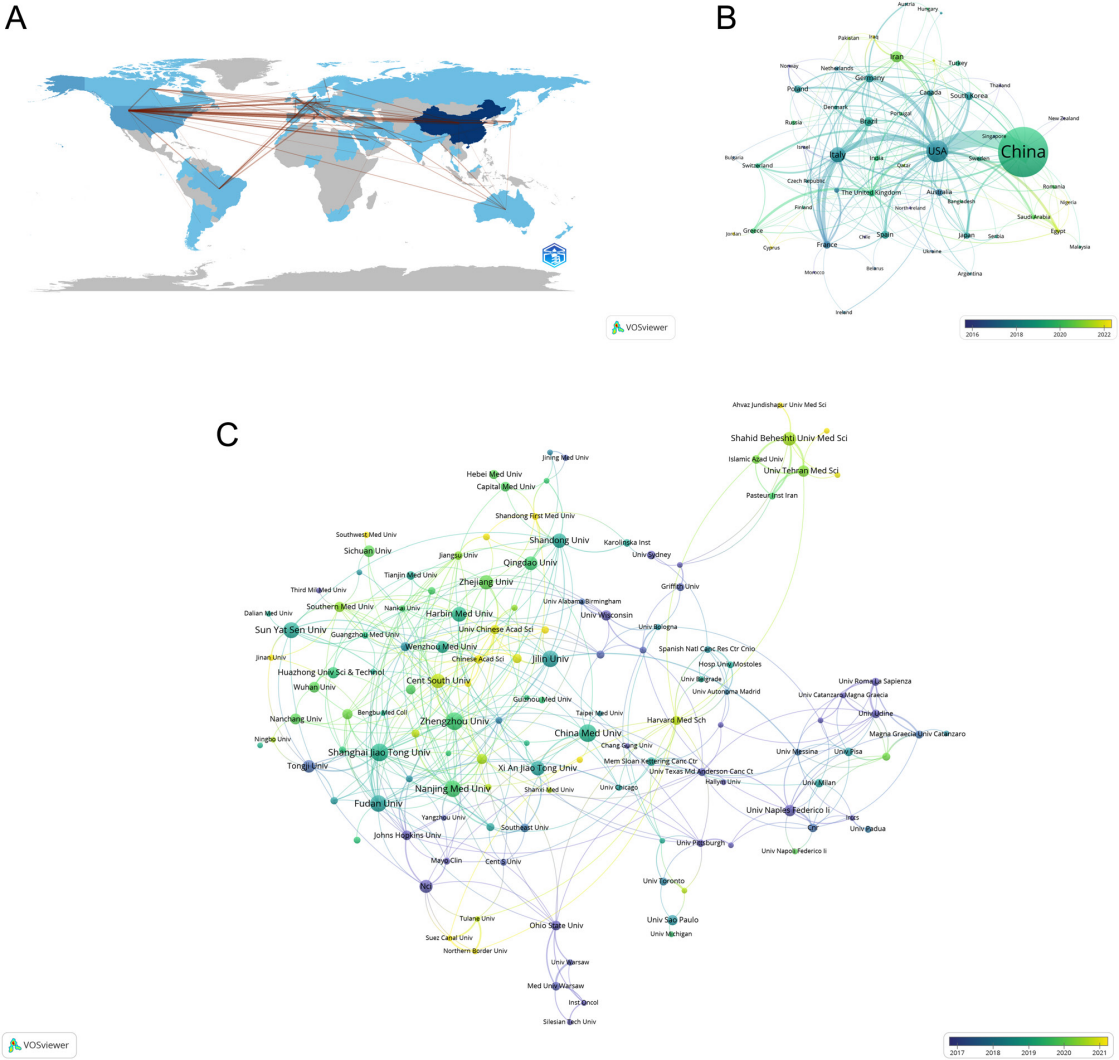
Geographical and institutional distribution in thyroid cancer epigenetics research. (A) Global research landscape indicating countries/regions involved with collaborative links representing cross-national cooperation. (B) Analysis of collaborative network visualization of countries/regions in VOSviewer according to average publication year (APY), depicting countries/regions with document counts exceeding one. Nodes transition from purple to yellow to indicate more recent entries, with size reflecting publication frequency. (C) Analysis of the number of articles published by institutions in recent years. The figure shows the institutions with more than six documents, where nodes follow the same color and size conventions as in (B).

The study spans 65 countries and 2081 institutions. The top ten contributing nations predominantly include five European, three Asian, and two North American countries (Table 1). China leads with 1379 publications (52.77%), followed by the USA (288 publications, 11.02%), Italy (151 publications, 5.78%), and Iran (80 publications, 3.06%). China and the USA together account for nearly two-thirds (63.79%) of the total output. A network analysis (Fig. 3A) shows significant collaboration, notably between the USA and China, Italy, and Germany, as well as among European countries. The USA, with a higher centrality index of 0.47, indicates a more influential position in the research network, as opposed to China’s lower centrality index of 0.08. Temporal trends (Fig. 3B) demonstrate a shift in the epicenter of research, with China’s increasing publication volume in recent years (2019–2022) following earlier prominence in the USA and Europe (2016–2018).

**Table 1:** Top 10 countries and institutions with the most documents in thyroid cancer epigenetics research.

Rank	Country	Centrality	Count (%)	Institution	Centrality	Count (%)
1	China	0.08	1379 (52.77%)	China Medical University	0.01	60 (2.89%)
2	USA	0.47	288 (11.02%)	Shanghai Jiao Tong University	0.11	59 (2.84%)
3	Italy	0.08	151 (5.78%)	Zhengzhou University	0.05	58 (2.79%)
4	Iran	0.03	80 (3.06%)	Jilin University	0.07	53 (2.55%)
5	Brazil	0.01	57 (2.18%)	Nanjing Medical University	0.02	52 (2.50%)
6	Germany	0.12	51 (1.95%)	Fudan University	0.23	51 (2.46%)
7	South Korea	0.01	46 (1.76%)	Sun Yat Sen University	0.06	49 (2.36%)
8	Spain	0.10	43 (1.65%)	Harbin Medical University	0.02	43 (2.07%)
9	France	0.11	42 (1.61%)	Zhejiang University	0.04	40 (1.93%)
10	Poland	0.01	41 (1.57%)	Central South University	0.04	38 (1.83%)

Institutional contributions reveal Chinese institutions’ dominance, with China Medical University leading in document count (2.89%). Shanghai Jiao Tong University and Fudan University, with centrality indices of 0.11 and 0.23, respectively, play significant integrative roles within the research community. A collaborative network of 142 institutions, based on a publication threshold of 6, depicts close cooperation among select universities (Fig. 3C). China Medical University, despite its high publication count, shows lower partnership with other institutions (centrality of 0.01, Table 1).

### Journals and co-cited journals

**Figure 4:**
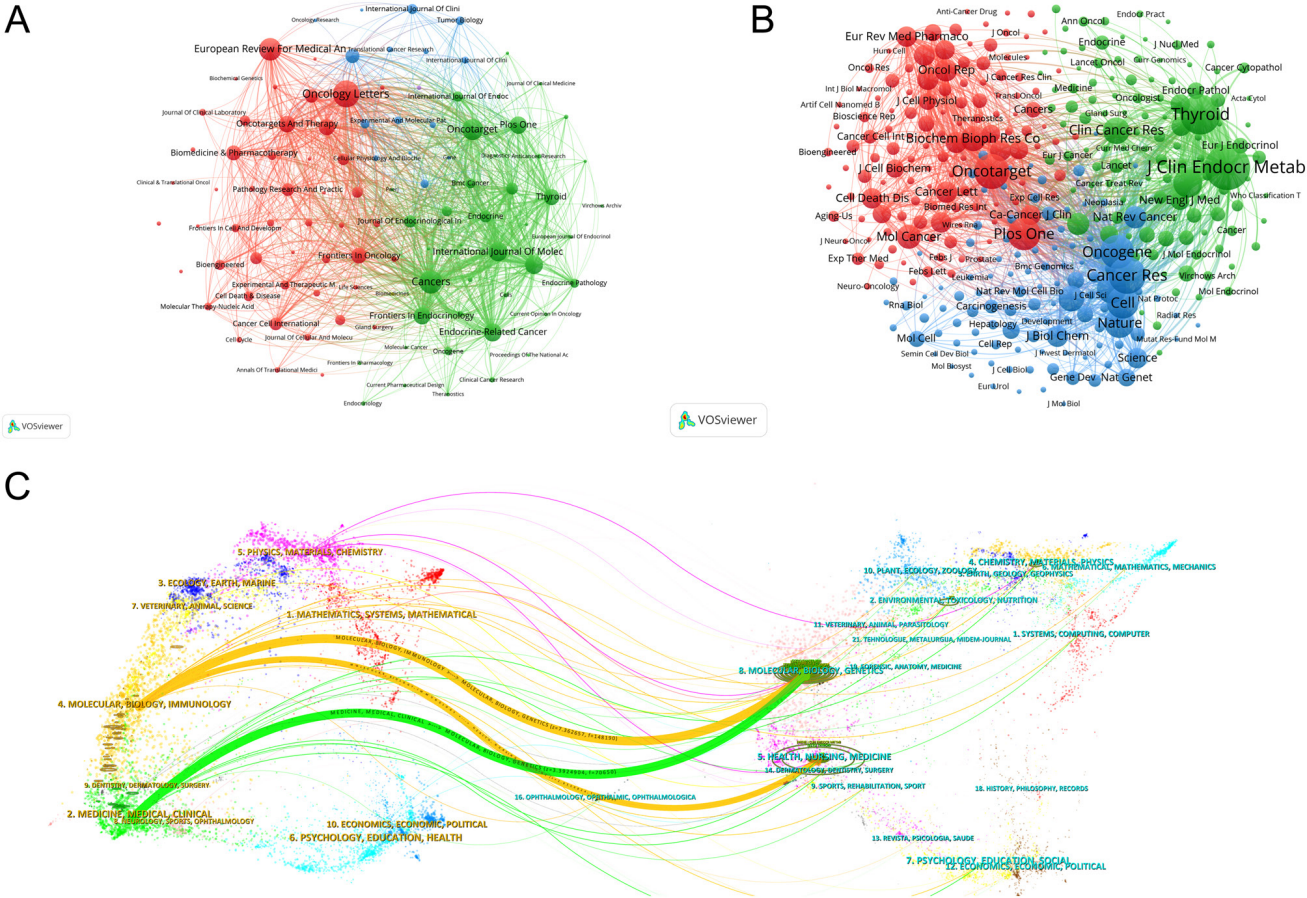
Journal analysis in thyroid cancer epigenetics. (A) Collaborative network visualization of journals in VOSviewer, highlighting journals with at least five documents. Node colors differentiate clusters, with size denoting publication frequency. (B) Analysis of collaborative network visualization of journals’ citations in VOSviewer. Each circle represents an journal, the circle size indicates the number of co-citations in that journal, the larger the circle, the higher the number of co-citations, the lines between the circles indicate the connections between journals, and the connection networks of different colors indicate the collaborative clusters between different journals. Different colors represent different clusters. (C) The dual-map overlay of journals. Citing journals are on the left, cited journals are on the right, and colored paths indicate citation relationships.

The concentration of research in specific scientific journals is evident (Table 2, Fig. 4A). *Oncology Letters*, with 59 publications (2.65% of total), leads, followed by *Cancers* and *European Review for Medical and Pharmacological Sciences*. These journals, with impact factors (IFs) ranging from 2.9 to 5.8, are predominantly ranked in the first and second quartiles of the JCR 2022, reflecting their influence.

**Table 2:** Top 10 journals publishing on epigenetics in thyroid cancer.

Rank	Journal	Publications (%)	IF (JCR 2022)	JCR quartile
1	*Oncology Letters*	59 (2.65%)	2.9	Q3
2	*Cancers*	50 (2.24%)	5.2	Q2
3	*European Review For Medical And Pharmacological Sciences*	48 (2.15%)	3.3	Q2
4	*Oncotarget*	44 (1.97%)	5.168	Q1
5	*International Journal of Molecular Sciences*	40 (1.80%)	5.6	Q1
6	*Journal of Clinical Endocrinology & Metabolism*	37 (1.66%)	5.8	Q1
7	*Endocrine-Related Cancer*	37 (1.66%)	3.9	Q2
8	*PLOS One*	36 (1.62%)	3.7	Q2
9	*Frontiers In Endocrinology*	35 (1.57%)	5.2	Q1
10	*Oncotargets And Therapy*	34 (1.53%)	4	Q2

Co-citation analysis reveals *Journal of Clinical Endocrinology & Metabolism* as the most cited journal (4059 citations), followed by *Thyroid* (3185 citations) and *Cancer Research* (2731 citations), underscoring their pivotal roles in disseminating epigenetics research (Fig. 4B, Table 3). Top-cited journals such as *Nature* and *Cell* have high impact factors (64.8 and 64.5, respectively), illustrating the correlation between high impact factors and citation frequency.

**Table 3:** Top 10 journals for co-citation of epigenetics in thyroid cancer.

Rank	Cited journal	Citations	IF (JCR 2022)	JCR quartile
1	*Journal of Clinical Endocrinology & Metabolism*	4059	5.8	Q1
2	*Thyroid*	3185	6.6	Q1
3	*Cancer Research*	2731	11.2	Q1
4	*Oncotarget*	2477	5.168	Q1
5	*Oncogene*	2248	8	Q1
6	*PLOS One*	2170	3.7	Q2
7	*Cell*	2046	64.5	Q1
8	*Nature*	1830	64.8	Q1
9	*PNAS*	1736	11.1	Q1
10	*Endocrine-Related Cancer*	1707	3.9	Q2

The dual-map overlay of journals in Fig. 4C illustrates the relationship between citing and cited journals. Articles within the Molecular/Biology/Genetics domain predominantly receive citations from works in the Molecular/Biology/Immunology and Medicine/Medical/Clinical domains. Similarly, articles in the Health/Nursing/Medicine domain are frequently cited by publications in the Molecular/Biology/Immunology domain. This mapping effectively represents the interconnectedness between citing and cited journals in various domains.

### Authors and co-cited authors

**Figure 5:**
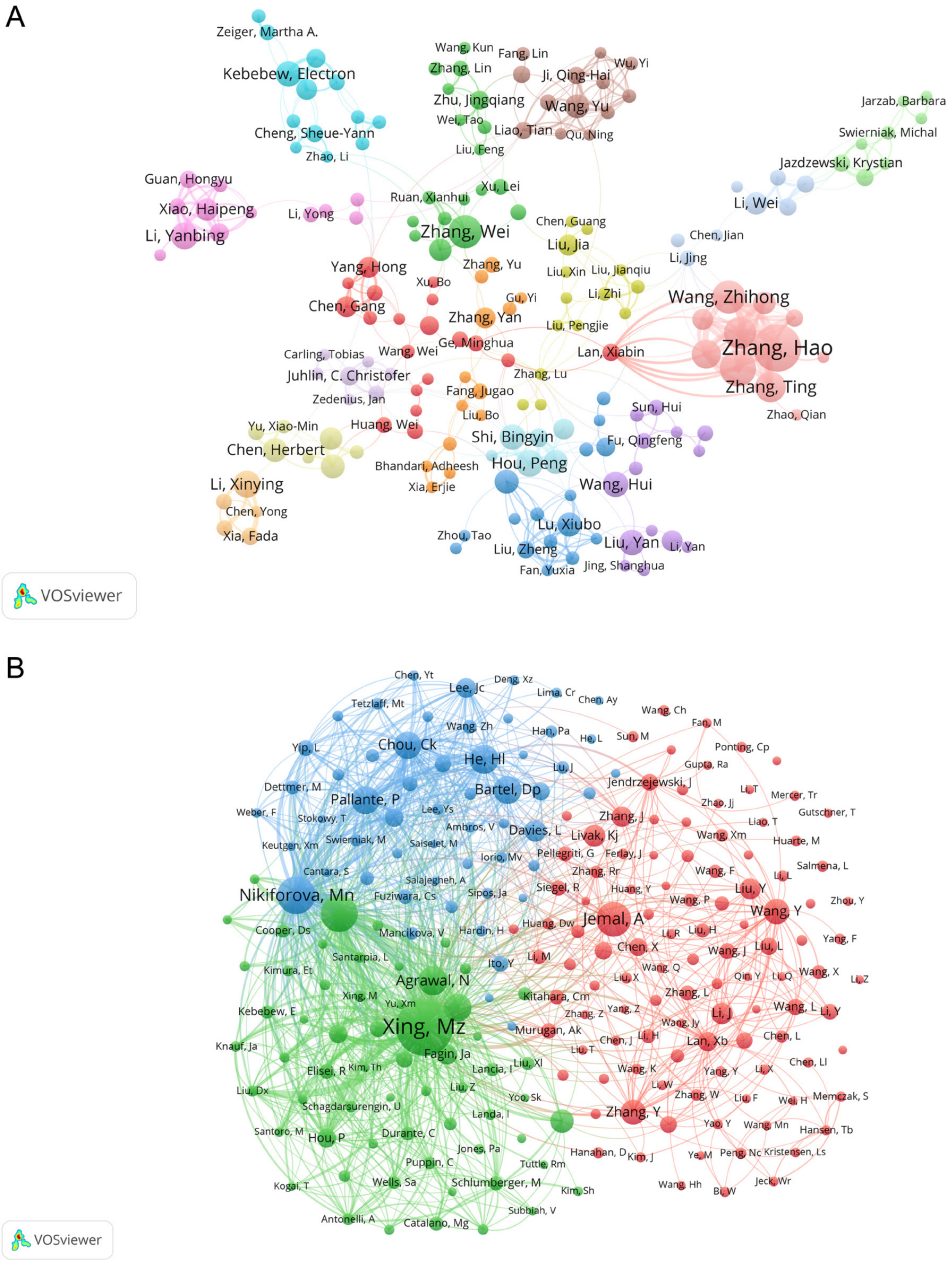
Author collaboration in thyroid cancer epigenetics. (A) VOSviewer’s network visualization showcasing authors with over five publications, with node colors identifying distinct clusters and size indicating publication counts. (B) Analysis of collaborative network visualization of authors’ citations in VOSviewer. Each circle represents an author, the circle size indicates the number of co-citations of that author’s published articles, the larger the circle, the higher the number of co-citations, the lines between the circles indicate the connections between authors, and the connection networks of different colors indicate the collaborative clusters between different authors. Different colors represent different clusters.

The bibliometric analysis mapped the epigenetics research landscape in thyroid cancer, pinpointing both the most prolific authors and those frequently co-cited (Fig. 5A and B). Table 4 displays the top 10 authors ranked by publication count, with H Zhang leading with 28 publications. Remarkably, seven of these prominent authors are based in China, underscoring the nation's considerable contributions to the field. Co-citation data identify M Xing as the most influential author, accruing 692 citations, a testament to his substantial impact in this area. Predominantly, these co-cited authors are based in the USA, with one notable exception from Italy.

**Table 4:** Top 10 authors and co-cited authors in thyroid cancer epigenetics.

Rank	Authors	Location	Count	Co-cited authors	Location	Citations
1	H Zhang	China	28	M Xing	USA	692
2	W Sun	China	20	M N Nikiforova	USA	386
3	A Fusco	Italy	19	Y E Nikiforov	USA	372
4	M Hedayati	Iran	19	A Jemal	USA	346
5	D Russo	Italy	19	N Agrawal	USA	271
6	Z Wang	China	18	H He	USA	271
7	W Zhang	China	18	P Pallante	Italy	257
8	W Dong	China	17	B R Haugen	USA	256
9	T Zhang	China	16	D P Bartel	USA	256
10	L He	China	15	C Chou	USA	248

### Co-cited references and references with citation bursts

Our study successfully pinpointed the most influential works in thyroid cancer epigenetics, as reflected by co-citation frequencies. Table 5 lists the top 10 co-cited references, with N Agrawal’s 2014 *Cell* article, ‘Integrated genomic characterization of papillary thyroid carcinoma’, leading the pack with 254 citations. This foundational work has profoundly influenced subsequent research in the field.

**Table 5:** Top 10 co-cited references in thyroid cancer epigenetics research.

Rank	Title	Citations	Article type	Year	First author	Journal
1	Integrated genomic characterization of papillary thyroid carcinoma	254	Original article	2014	N Agrawal	*Cell*
2	2015 American Thyroid Association Management Guidelines for adult patients with thyroid nodules and differentiated thyroid cancer: The American Thyroid Association Guidelines Task Force on thyroid nodules and differentiated thyroid cancer	211	Guideline	2016	B R Haugen	*Thyroid*
3	The role of microRNA genes in papillary thyroid carcinoma	197	Original article	2005	H He	*PNAS*
4	Molecular pathogenesis and mechanisms of thyroid cancer	190	Review	2013	M Xing	*Nature Reviews Cancer*
5	MicroRNA expression profiling of thyroid tumors: biological significance and diagnostic utility	159	Original article	2008	M N Nikiforova	*Journal of Clinical Endocrinology & Metabolism*
6	MicroRNAs: genomics, biogenesis, mechanism, and function	153	Review	2004	D P Bartel	*Cell*
7	Cancer statistics, 2009	145	Original article	2009	A Jemal	*CA: A Cancer Journal for Clinicians*
8	Thyroid cancer	141	Review	2016	M E Cabanillas	*Lancet*
9	MicroRNA deregulation in human thyroid papillary carcinomas	131	Original article	2006	P Pallante	*Endocrine-Related Cancer*
10	Global cancer statistics	126	Original article	2011	A Jemal	*CA: A Cancer Journal for Clinicians*

**Figure 6:**
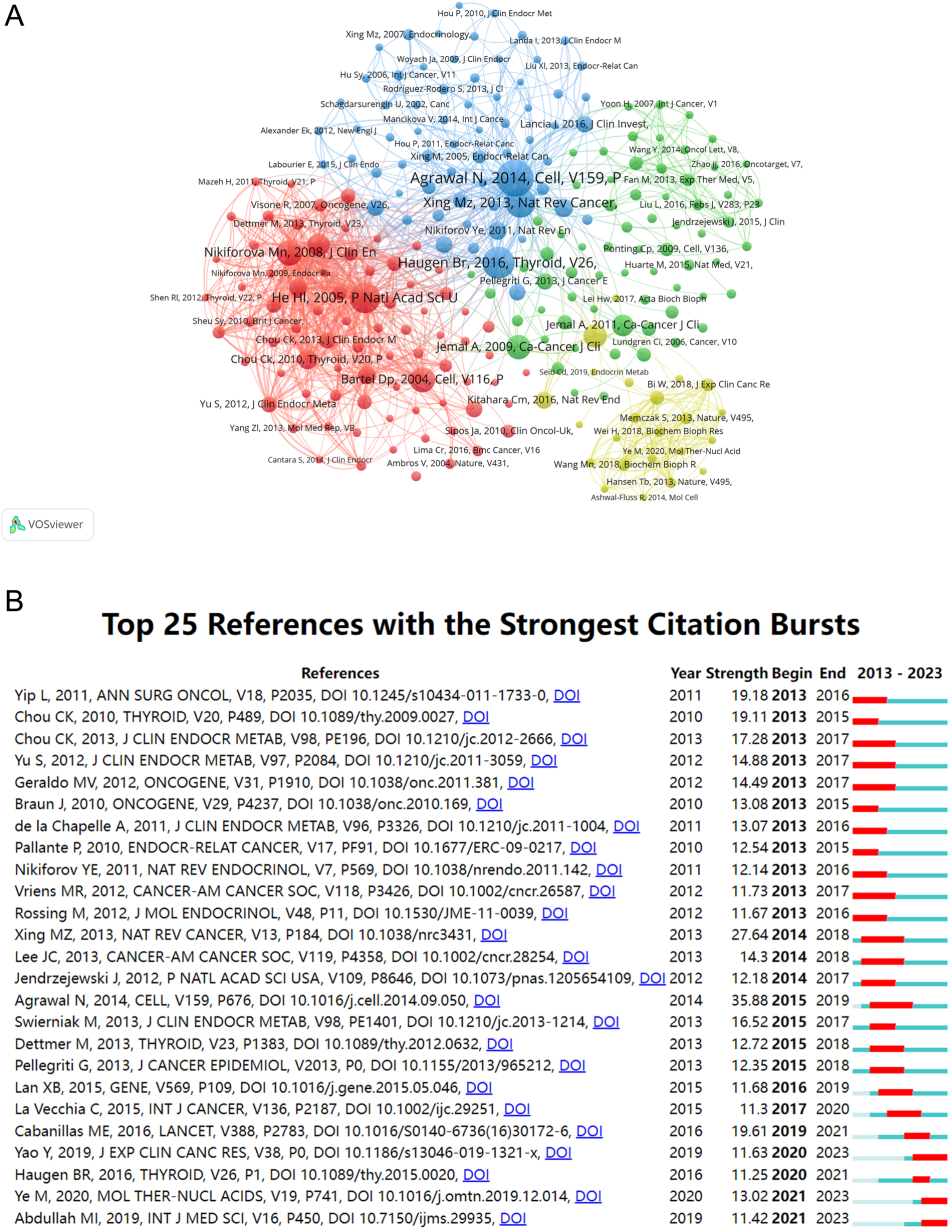
Reference analysis in thyroid cancer epigenetics. (A) Network of co-cited references with each circle representing a reference. Circle size corresponds to co-citation counts, lines indicate inter-reference connections, and colors signify collaborative clusters. (B) CiteSpace visualization map of top 25 references with the strongest citation bursts within the field.

These leading articles, predominantly published in distinguished journals such as *Cell* and *CA: A Cancer Journal for Clinicians*, each contributing two articles to the top ten (Table 5, Fig. 6A), cover a diverse array of epigenetic topics, including regulatory mechanisms, molecular underpinnings, and their implications in both tumor suppression and cancer therapy.

The ‘citation burst’ analysis highlighted articles that experienced a rapid increase in citations over a specific period, signaling the emergence of significant research topics (25). Our analysis identified the top 25 references with notable citation bursts (Fig. 6B), indicating a burgeoning interest in this field from 2013 to 2023. The most cited paper, N Agrawal’s ‘Integrated genomic characterization of papillary thyroid carcinoma’, demonstrated the strongest citation burst (strength = 35.88), with peak citations occurring between 2015 and 2019. M Xing *et al.*’s work, ‘Molecular pathogenesis and mechanisms of thyroid cancer’, published in *Nature Reviews Cancer*, ranked second in citation bursts (strength = 27.64), spanning from 2014 to 2018. The burst strengths for these 25 references ranged from 11.25 to 35.88, with durations spanning 1–4 years.

### Hotspots and frontiers

Through VOSviewer and CiteSpace, we analyzed 3788 author keywords across 2228 documents, identifying 116 keywords that appeared in at least seven documents each.

**Figure 7:**
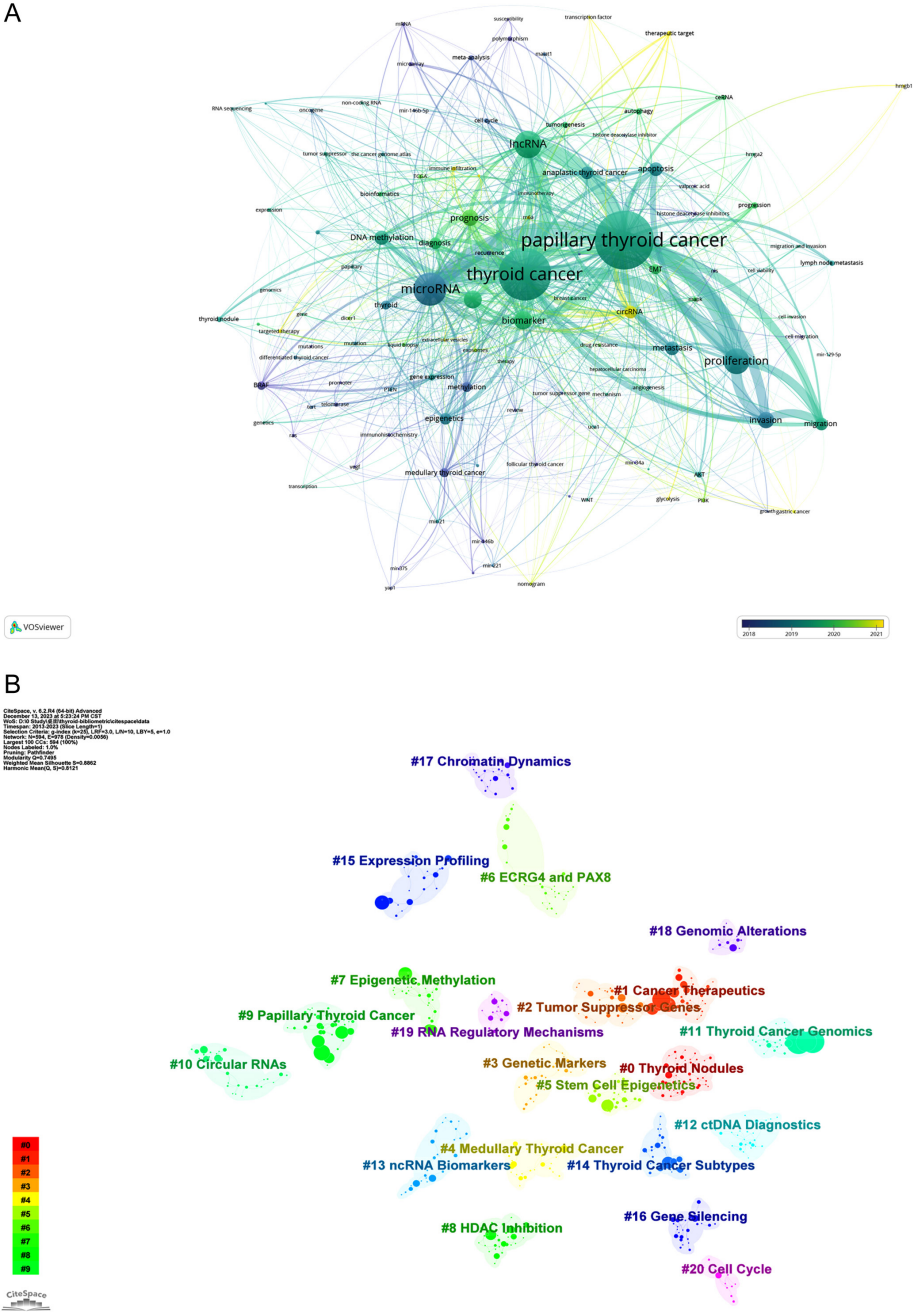
Keyword Analysis in Thyroid Cancer Epigenetics Research. (A) Keyword collaboration network visualized in VOSviewer by APY, focusing on keywords with over seven documents. Nodes change from purple to yellow reflecting their emergence in literature, with size indicating keyword prominence. (B) Clustering analysis of the keyworks network based on CiteSpace in Thyroid Cancer Epigenetics Research.

Our bibliometric analysis, integrating keyword co-occurrence and temporal progression mapping, illuminated the evolving trends in thyroid cancer epigenetics research. ‘Thyroid cancer’ emerged as a central node in the co-occurrence network, frequently cited across the literature. Adjacent to this central node, significant terms such as ‘microRNA’, ‘lncRNA’, ‘circRNA’, ‘DNA methylation’, and ‘EMT’ were prominent, highlighting their relevance in the field (Fig. 7A). The temporal progression map shed light on emerging research areas, with terms such as ‘circRNA’, ‘immune infiltration’, and ‘glycolysis’ colored in a yellowish hue, denoting their status as frontier topics (Fig. 7A). In contrast, foundational terms such as ‘BRAF’ and ‘ras’ were depicted in purple, providing historical context to these evolving trends.

Cluster analysis via CiteSpace yielded 23 clusters, 21 of which are detailed in Fig. 7B, ranging from ‘Thyroid Nodules’ to ‘Cell Cycle’. Clusters 21 and 22 were omitted due to their small size. The modularity (*Q*) value above 0.3 and silhouette (*S*) value above 0.7, obtained in our analysis (*Q* = 0.75, *S* = 0.89), affirm the robustness and significance of these clusters.

## Discussion

This study utilized VOSviewer, CiteSpace, and the ‘bibliometrix’ R package to analyze 2228 articles on thyroid cancer epigenetics, uncovering significant spatial, temporal, and influential trends within the field. Our findings underscore the increasingly recognized importance of epigenetic mechanisms in understanding and treating thyroid cancer.

### General information

The sustained publication volume, despite a recent slight decline, indicates a robust interest in this domain. This trend reflects the ongoing advancements that are shaping innovative diagnostic and therapeutic strategies.

### Geographical and institutional distribution

China and the USA lead in research output, with significant European contributions complementing their efforts. China's extensive output, attributed to substantial research investment, contrasts with its lower centrality in global collaborations, suggesting an emerging, yet maturing research domain. This burgeoning collaboration across continents is likely enhancing the quality of research, as depicted in our network analyses.

### Status and quality of journals, authors, and references

In thyroid cancer epigenetics research, M Xing emerges as the most cited author with 692 citations, followed by M N Nikiforova and Y E Nikiforov. M Xing’s influential 2013 review article in *Nature Reviews Cancer*, ‘Molecular pathogenesis and mechanisms of thyroid cancer’, has been pivotal in advancing understanding of epigenetics in thyroid cancer. It explores the significant role of epigenetic alterations, including DNA methylation and histone modification patterns, in disease progression. The paper also highlights the emerging importance of non-coding RNAs, such as miRNAs, lncRNAs, and circRNAs, in the epigenetic landscape of thyroid cancer. Furthermore, it examines the complex interplay between epigenetic changes and genetic mutations in the disease (10). M Xing’s substantial contributions have laid a solid foundation for ongoing research in this area. His prominent citation count reflects his profound influence and recognition within the scientific community, underscoring his vital role in shaping thyroid cancer epigenetics research. The significant citation counts for authors such as M N Nikiforova and Y E Nikiforov illustrate their extensive research into miRNA expression patterns in various thyroid tumor types, including those with specific oncogenic mutations. Their work investigates the potential of miRNA profiling for the preoperative diagnosis of thyroid nodules (26). This bibliometric analysis is invaluable for guiding future collaborative efforts, identifying key researchers and potential collaborators, and assisting new researchers in quickly familiarizing themselves with the foundational literature and ongoing discussions in the field.

The concept of co-cited references, which are references cited concurrently by multiple publications, serves as a foundation within a specific study area (27). In this analysis, the top 10 co-cited references provide essential insights into epigenetics in thyroid cancer. Among these, N Agrawal’s highly co-cited work, ‘Integrated genomic characterization of papillary thyroid carcinoma’, reveals recurrent mutations in key epigenetic regulatory genes, such as MLL, ARID1B, and MLL3, highlighting their potential role in thyroid cancer development. The study’s integrated approach sheds light on the complex interplay between epigenetic regulation, miRNA expression, and overall gene expression, particularly focusing on miR-21, which is epigenetically regulated and linked to aggressive tumor behavior. This research emphasizes the criticality of epigenetic mechanisms and miRNA dysregulation in thyroid cancer, prompting further exploration of their functional roles and therapeutic potential (28). Additionally, B R Haugen’s 2016 guideline in *Thyroid*, ‘2015 American Thyroid Association Management Guidelines for Adult Patients with Thyroid Nodules and Differentiated Thyroid Cancer’, discusses the implications of epigenetic changes in thyroid cancer progression and development. It underscores the need for ongoing research into the mechanisms behind these epigenetic alterations and their potential as biomarkers for early detection, prognosis, and therapy in thyroid cancer (29).

### Hotspots and frontiers

Our analysis identified several distinct clusters in thyroid cancer epigenetics research (Fig. 7B), which can be broadly categorized into the following themes:

### Molecular mechanisms and biomarkers in thyroid cancer

#### DNA methylation and its implications

DNA methylation, primarily occurring at CpG islands within promoter regions, has been extensively studied in thyroid cancer (30). Extensive research has demonstrated that this modification can lead to silencing of tumor suppressor genes, playing a pivotal role in thyroid carcinogenesis (31). The landmark study by M Xing *et al.* is a notable example, highlighting the hypermethylation of the TSHR gene's promoter region and its correlation with reduced expression in thyroid cancer tissues. This change promotes tumor growth and proliferation, illustrating the intricate mechanisms by which epigenetic alterations can drive cancer progression (32). Furthermore, global hypomethylation, observed in various thyroid cancer studies, contributes to genomic instability, a hallmark of many cancers (33, 34). This phenomenon can lead to increased mutation rates and chromosomal abnormalities, further driving the disease’s progression (35). Recent advancements in high-throughput sequencing technologies have revolutionized the understanding of these methylation patterns (36). For instance, the study by Iqbal *et al.* used whole-genome bisulfite sequencing to map the methylome of thyroid cancer cells, uncovering novel hypermethylated regions that could serve as potential biomarkers for early detection and prognosis (37).

#### Histone modifications in thyroid cancer

Histone modifications, including acetylation, methylation, phosphorylation, and ubiquitination, are crucial for regulating gene expression and chromatin structure in thyroid cancer (38). The aberrant expression of histone deacetylases (HDACs) and histone methyltransferases has been closely linked with the progression and aggressiveness of thyroid cancer (39). For example, the study by Borbone *et al.* demonstrated that overexpression of HDAC in anaplastic thyroid carcinoma is associated with poor prognosis and aggressive tumor behavior (40). These findings underline the importance of histone modifiers as potential therapeutic targets in thyroid cancer. Another significant modification in this context is the methylation of histone H3 on lysine 27 (H3K27me3), often associated with gene silencing and chromatin condensation (41). The enhancer of zeste homolog 2 (EZH2), a key enzyme responsible for catalyzing H3K27me3, has been reported to be overexpressed in advanced stages of thyroid cancer . Fu *et al.*s research indicated that EZH2 plays a vital role in thyroid cancer progression, suggesting its potential as a target for epigenetic therapy (42).

#### Role of non-coding RNAs in thyroid cancer epigenetics

The role of non-coding RNAs, particularly miRNAs and lncRNAs, has become increasingly evident in the epigenetic regulation of thyroid cancer. These molecules can regulate gene expression post-transcriptionally and are integral to the epigenetic landscape of thyroid cancer (43). Endogenous miRNAs play a pivotal role in cellular processes including proliferation, differentiation, apoptosis, and autophagy (44). They are critical in the pathogenesis of thyroid cancer, either by upregulating oncogenes or downregulating tumor suppressor genes, thus influencing the disease’s onset and progression (45, 46). A recent breakthrough in this field is the discovery of the impact of miR-455-5p on the CXCL12/CXCR4 signaling pathway. This interaction has been shown to attenuate the proliferation of thyroid cancer cells and impede tumor growth, presenting a potential avenue for therapeutic intervention (47).

lncRNAs also play a significant role in thyroid cancer pathogenesis. For instance, the lncRNA HOTAIR has been implicated in thyroid cancer metastasis. The study by Wang *et al.* demonstrated that HOTAIR promotes the development and progression of thyroid cancer through inhibition of microRNA-1 and activation of CCND2 (48). Additionally, circRNAs have been recognized for their role in thyroid cancer epigenetics, particularly in papillary thyroid cancer (PTC). Studies have shown that certain circRNAs, such as hsa_circ_0039411, are upregulated in PTC and promote cancer cell growth, migration, and invasion, while inhibiting apoptosis (49).

#### Epigenetic biomarkers for diagnosis and prognosis

The identification of epigenetic biomarkers has profound implications for the diagnosis and prognosis of thyroid cancer. Various studies have proposed methylation signatures, specific histone modification patterns, and aberrant expression of non-coding RNAs as potential biomarkers (50). For example, recent research has identified a panel of DNA methylation patterns capable of distinguishing between benign and malignant thyroid nodules with high accuracy (51). Similarly, alterations in histone modification patterns, as demonstrated in several studies, have been proposed as markers of aggressive disease and poor prognosis (52). The expression profiles of miRNAs have also gained attention as diagnostic and prognostic tools in thyroid cancer. A notable study by Eman *et al.* developed a miRNA signature that can predict recurrence in papillary thyroid cancer patients, offering a new approach to patient management and treatment planning (53). Additionally, the importance of circulating non-tissue markers, such as cell-free DNA and VEGF pathway polymorphisms, is increasingly recognized in advanced disease management. The analysis of cell-free DNA provides insights into the genetic and epigenetic alterations associated with thyroid cancer, supporting its role in early detection and monitoring (54). Similarly, germline polymorphisms in the VEGF pathway have been linked to recurrence in non-advanced differentiated thyroid cancer, suggesting their potential to refine prognostic assessments and guide treatment decisions (55). Furthermore, the pioneering study by Hoque *et al.* introduced the detection of serum DNA methylation markers as a novel diagnostic tool for thyroid cancer. This approach represents one of the earliest instances of using non-tissue-based epigenetic markers, underscoring their potential to revolutionize the diagnostic landscape of thyroid cancer (56).

In addition to the previously discussed epigenetic biomarkers, recent advancements in the field of miRNAs have significantly enhanced our diagnostic capabilities for MTC. Notably, the Ferretti research group has demonstrated the potential of circulating miR-26b-5p and miR-451a as reliable non-invasive diagnostic biomarkers for MTC. Their study validated these miRNAs in both discovery and independent cohorts, illustrating their significant diagnostic performance and their capacity to be monitored post-surgery, thereby offering a novel approach to precision medicine in thyroid cancer management (57).

### Epigenetic heterogeneity in thyroid cancer subtypes

Thyroid cancer presents a diverse epigenetic landscape across its various subtypes, including PTC, follicular thyroid cancer (FTC), medullary thyroid cancer (MTC), and anaplastic thyroid cancer (ATC). This diversity is not only a reflection of the unique pathological features of each subtype but also signifies the potential for targeted epigenetic therapies (58).

PTC, the most common subtype, is often characterized by BRAF V600E mutations, significantly impacting its epigenetic landscape. Studies have shown that this mutation is associated with distinct methylation patterns, particularly in the promoter regions of tumor suppressor genes (59). For instance, studies have shown a significant increase in promoter methylation of the TSHR gene in BRAF-mutated PTC samples, leading to gene silencing and subsequent tumor progression. This finding highlights the importance of understanding the interplay between genetic mutations and epigenetic modifications in thyroid cancer (60). In FTC, RAS mutations are prevalent and have been found to correlate with specific histone modification patterns. These mutations are present in about 40–50% of FTC cases and are associated with changes in chromatin structure and gene expression (61). The RASSF1A tumor suppressor gene, a major isoform of the RASSF1 gene, is a key player in this context. It possesses a RAS association domain and functions in controlling cell cycle progression and apoptosis. The epigenetic regulation of RASSF1A and its impact on FTC pathogenesis remains a critical area of research (62). MTC displays a unique epigenetic profile, particularly in the context of RET mutations (63). Histone methyltransferases such as EZH2 and SMYD3 are key features in MTC, with increased expression observed much more frequently than in PTC or FTC (64). These findings suggest that MTC may require different therapeutic approaches, particularly those targeting specific epigenetic enzymes. ATC is the most aggressive subtype of thyroid cancer and exhibits extensive DNA methylation alterations. For example, a significant increase in thyroid transcription factor-1 DNA methylation levels has been reported in ATC compared to other subtypes. This extensive methylation contributes to the aggressive nature of ATC and its poor prognosis (65).

The study of epigenetic heterogeneity across thyroid cancer subtypes is crucial for developing personalized and effective treatment strategies. Understanding the unique epigenetic alterations in each subtype provides insights into their pathogenesis and potential therapeutic targets, paving the way for more precise and targeted interventions.

### Therapeutic implications of epigenetic alterations in thyroid cancer

#### Current epigenetic therapies in thyroid cancer

Treatment strategies for thyroid cancer typically include surgical resection, radioactive iodine therapy, and thyroid hormone suppression therapy (66). However, in advanced stages or when the cancer becomes refractory to these traditional treatments, therapeutic options become limited. Under these circumstances, kinase inhibitors such as lenvatinib play a critical role. While these inhibitors have been pivotal in managing radioiodine-refractory differentiated thyroid cancer, they are not curative and focus primarily on prolonging progression-free survival. Studies have shown that, although they significantly control disease progression, these treatments do not eradicate the cancer, emphasizing the necessity for continued therapeutic innovation and research (67, 68). This limitation underscores the urgent need for novel treatment approaches, including those targeting epigenetic modifications, which offer the potential to more effectively manage and potentially overcome the challenges presented by advanced thyroid cancers.

The integration of epigenetic therapies in thyroid cancer treatment represents a new frontier in oncology. The ongoing development and clinical trials of DNA methyltransferase inhibitors (DNMTis) and histone deacetylase inhibitors (HDACis) are particularly promising (69, 70). In addition to exploring the clinical potential of epigenetic therapies in thyroid cancer treatment, considerable preclinical evidence supports their efficacy. For instance, studies on DNMTis such as Decitabine have demonstrated their ability to reactivate silenced tumor suppressor genes in thyroid cancer cell lines. This reactivation leads to inhibited tumor growth and reduced metastatic potential, as observed in several in vitro and in vivo models (71). Similarly, HDACis such as SAHA have shown promising results in preclinical trials, where they increase the expression of the Na+/iodine symporter in thyroid cancer cells, enhancing the effectiveness of radioiodine therapy in models that are typically resistant to this treatment (72). These preclinical studies not only underscore the therapeutic potential of these epigenetic drugs but also provide a foundation for future clinical trials aimed at integrating these agents into standardized treatment protocols for thyroid cancer.

#### Emerging therapies and mechanisms

Recent research has focused on new classes of epigenetic drugs, such as bromodomain and extra-terminal (BET) inhibitors and BRD4 inhibitors (73). These drugs target proteins that recognize acetylated histones and disrupt transcriptional networks crucial for thyroid cancer cell survival and proliferation (74, 75). JQ1 and I-BET762, two BRD4 inhibitors, have shown efficacy in halting the cell cycle in ATC cells by targeting key cellular pathways, suggesting their potential in future clinical trials (73, 76).

#### Challenges in drug delivery and specificity

A significant challenge in epigenetic therapy is achieving targeted drug delivery without affecting normal tissue (77). The non-specific action of current epigenetic drugs often leads to off-target effects and toxicity, necessitating the development of more targeted delivery systems (78, 79). Overcoming drug resistance, another major hurdle, requires innovative approaches (80). Combination therapy, using epigenetic drugs alongside standard chemotherapy, targeted drugs, or immunotherapy, has shown promise in addressing this challenge (76, 81, 82).

While the potential of epigenetic therapies in thyroid cancer treatment is promising, it is important to note that these approaches are currently considered emerging. As outlined in the recent ESMO Clinical Practice Guidelines on thyroid cancer management, epigenetic therapies have not yet been fully endorsed by regulatory agencies and are not included in the standard treatment guidelines. This is primarily due to the need for further clinical evidence to validate their efficacy and safety. The guidelines emphasize the importance of advancing research and incorporating findings into clinical practice to ensure these innovative therapies can be integrated into routine care once proven effective (83).

The therapeutic implications of epigenetic alterations in thyroid cancer are vast and complex. While current therapies show promise, ongoing research and development are essential for advancing treatment strategies. The integration of these therapies into personalized treatment regimens holds great potential for improving outcomes in thyroid cancer patients. As highlighted by Marotta *et al.*, the prognostic utility of genetic markers such as BRAFV600E, TERT, and TP53 mutations presents a significant challenge for clinicians striving to optimize treatment strategies (84). Addressing these challenges is key to transitioning towards personalized medicine, which promises more targeted and effective interventions.

### Novel technologies and methodologies in epigenetics research

Advancements in technology and methodology have dramatically influenced the study of epigenetics in thyroid cancer, providing new insights into its molecular basis and opening avenues for novel diagnostic and therapeutic approaches.

#### Next-generation sequencing

The advent of next-generation sequencing has been a game changer in epigenetics, enabling genome-wide analysis of epigenetic modifications. Techniques such as whole-genome bisulfite sequencing and chromatin immunoprecipitation sequencing have provided unprecedented insights into DNA methylation patterns and histone modification landscapes (85, 86). These methods are pivotal in identifying epigenetic markers associated with thyroid cancer, offering new avenues for targeted therapies.

#### Single-cell epigenetics

Single-cell analyses have brought to light the cellular heterogeneity within thyroid tumors. Studies utilizing single-cell RNA sequencing have revealed diverse cellular microenvironments and tumor heterogeneity in thyroid cancer, correlating with varying levels of aggressiveness and treatment response (87). This information is critical for developing personalized treatment strategies tailored to individual tumor profiles.

#### CRISPR/Cas9-based epigenome editing

The CRISPR/Cas9 technology has enabled targeted studies of gene function in thyroid cancer (88, 89). Researchers have used this technology to identify key genes and pathways involved in tumor growth and progression, offering potential avenues for gene-based therapies. For instance, CRISPR/Cas9 has been used to identify essential glycolytic enzymes in thyroid cancer cells, providing insights into metabolic pathways driving tumor growth (90).

#### Integrating omics data in thyroid cancer research

The integration of omics approaches, encompassing genomic, transcriptomic, and epigenomic data, has been pivotal in revealing new perspectives of thyroid cancer (91, 92). A notable study utilizing both whole exome sequencing and RNA sequencing of PTC samples uncovered a novel molecular signature. This signature effectively categorizes PTC patients into two distinct subtypes, each characterized by unique clinicopathological features, genomic alterations, gene expression profiles, immune microenvironment patterns, and responses to immunotherapy (93). Such comprehensive analyses have been instrumental in shedding light on intricate aspects of thyroid cancer subtypes, enhancing our understanding of their diverse clinicopathological manifestations, molecular underpinnings, and potential therapeutic responses.

### Computational epigenetics

In the realm of thyroid cancer research, computational epigenetics has emerged as a crucial tool for managing and interpreting complex datasets (94, 95). Machine learning models, in particular, have shown significant promise. They are increasingly being employed not only for basic genomic research but also for applications in diagnosis, drug discovery, and epigenetic analysis (96). The potential of these computational approaches extends to the development of innovative diagnostic tools and new therapeutic strategies, poised to transform the landscape of thyroid cancer management in the foreseeable future.

### Future directions

Based on our bibliometric analysis, we propose several avenues for future research in epigenetics in thyroid cancer:

1. **Deeper mechanistic insights:** Continued research is essential to elucidate the complex molecular mechanisms behind epigenetic alterations in thyroid cancer, including the identification of key regulators and signaling pathways. This knowledge is crucial for developing novel epigenetic therapies.
2. **Subtype-specific epigenetics:**Future studies should aim to comprehensively profile epigenetic variations across different thyroid cancer subtypes. Large-scale research integrating molecular profiling with clinical data is imperative for understanding the clinical implications of these profiles in targeted therapies and personalized medicine.
3. **Epigenetic therapeutic strategies:**Ongoing studies are needed to evaluate the effectiveness and safety of epigenetic drugs in treating thyroid cancer, including research on combination therapies and new epigenetic targets. Well-designed clinical trials with extensive follow-up are vital to confirm the role of epigenetic therapy in thyroid cancer management.
4. **Integration of multi-omics data:**Integrating epigenetic data with genomic, transcriptomic, and proteomic information can provide a comprehensive view of the molecular alterations in thyroid cancer. Such integrative multi-omics studies are critical for understanding the interplay between genetic, epigenetic, and environmental factors in the disease.
5. **Translational applications:**Future research should concentrate on applying epigenetic findings in clinical settings, developing diagnostic tests, prognostic models, and therapeutic strategies based on epigenetics. Collaborative efforts across research, clinical, and industry sectors are essential for the successful translation of epigenetic approaches into the management of thyroid cancer.

In light of the data presented, we must consider the potential advancements in epigenetics and their implications for managing thyroid cancer. This expert opinion elucidates how these emerging technologies could revolutionize clinical practice across various stages of disease management. Epigenetic markers, including DNA methylation profiles and histone modification patterns, are set to improve the accuracy of early detection methods, enabling precise differentiation between benign and malignant nodules. In prognostics, integrating epigenetic data offer deeper insights into tumor aggressiveness and potential treatment responses, thereby personalizing care. Monitoring changes in epigenetic signatures during follow-up could serve as a non-invasive indicator of disease recurrence, enhancing patient monitoring post-treatment. Additionally, in the management of advanced disease, therapies targeting DNA methyltransferases and histone deacetylases offer new treatment avenues, particularly for patients resistant to conventional methods. These advancements underscore the need for ongoing research and collaboration between clinicians and researchers to unlock the full potential of epigenetics in improving thyroid cancer outcomes.

### Limitations

This study, while providing a thorough visual bibliometric analysis of epigenetics in thyroid cancer research until 1 November 2023, has its limitations. Bibliometric analyses, by their nature, offer a snapshot in time and therefore need regular updates to reflect the field’s evolving dynamics. Our reliance solely on the WOSCC might have overlooked pertinent studies from other databases such as PubMed, Cochrane Library, and Google Scholar. The emphasis on English-language publications also introduces a language bias, potentially missing critical research in other languages. Dataset inconsistencies, including variable institutional naming, might have affected the precision of our analysis. Moreover, acknowledging the strengths and limitations of the bibliometric tools we employed – VOSviewer, CiteSpace, and the R package ‘bibliometrix’ – is essential. While these tools are robust, they may not capture the complete range of available data, possibly resulting in analytical gaps.

## Conclusion

Our bibliometric analysis provides a comprehensive overview of the current state of research in thyroid cancer epigenetics, identifying key themes, hotspots, and directions for future research. The findings highlight the significant role of epigenetics in uncovering the molecular mechanisms, biomarkers, and therapeutic implications of thyroid cancer. The potential to develop epigenetic-based diagnostic tools, prognostic models, and therapeutic approaches is substantial, offering promising avenues to enhance patient care and outcomes. This study enriches the growing knowledge base in thyroid cancer epigenetics and underscores the importance of continued research to maximize the benefits of epigenetics in managing this complex disease. Delving deeper into the intricate mechanisms of epigenetic alterations can lead to groundbreaking therapeutic interventions and personalized treatments, thereby improving patient outcomes in thyroid cancer.

## Declaration of interest

The authors declare that there is no conflict of interest that could be perceived as prejudicing the impartiality of the study reported.

## Funding

This work did not receive any specific grant from any funding agency in the public, commercial, or not-for-profit sector.

## Ethics approval

This study was prepared in accordance with the Committee on Publication Ethics (COPE) guidelines to respect third parties’ rights such as copyright and/or moral rights. Ethical approval was not required to conduct this project, as data are not individualized and primary data were not collected.

## Consent for publication

All authors have read and approved the content and agree to submit the final version of the manuscript for consideration and publication in this journal.

## Availability of data and materials

The datasets from the current study are available from the corresponding author upon reasonable request.

## Author contribution statement

Study conception and design: PW; data collection and data analysis: HL; preparation of the first draft of the manuscript: HL; critical revision of the manuscript for important intellectual content: PW. All authors read and approved the final version of the manuscript.
